# Shanghai *Neisseria gonorrhoeae* Isolates Exhibit Resistance to Extended-Spectrum Cephalosporins and Clonal Distribution

**DOI:** 10.3389/fmicb.2020.580399

**Published:** 2020-10-06

**Authors:** Yuan Dong, Yang Yang, Ying Wang, Irene Martin, Walter Demczuk, Weiming Gu

**Affiliations:** ^1^Shanghai Skin Disease Hospital, Shanghai, China; ^2^Shanghai Municipal Center for Disease Control and Prevention, Shanghai, China; ^3^Public Health Agency of Canada, National Microbiology Laboratory, Winnipeg, MB, Canada

**Keywords:** *Neisseria gonorrhoeae*, extended-spectrum cephalosporins, multidrug resistance, resistance determinants, phylogenetic analysis

## Abstract

The emergence of *Neisseria gonorrhoeae* strains with resistance (R) to extended-spectrum cephalosporins (ESCs^R^) represents a public health threat of untreatable gonococcal infections. This study was designed to determine the prevalence and molecular mechanisms of ESC^R^ of Shanghai *N. gonorrhoeae* isolates. A total of 366 *N. gonorrhoeae* isolates were collected in 2017 in Shanghai. Susceptibility to ceftriaxone (CRO), cefixime (CFM), azithromycin (AZM), ciprofloxacin (CIP), spectinomycin, penicillin, and tetracycline was determined using the agar dilution method. A subset of 124 isolates was subjected to phylogenetic analysis for nine antimicrobial resistance-associated genes, i.e., *penA*, *porB*, *ponA*, *mtrR*, 23S rRNA, *gyrA*, *parC*, 16S rRNA, and *rpsE*. Approximately 20.0% of the isolates exhibited CFM^R^ [minimum inhibitory concentration (MIC) >0.125 mg/L], and 5.5% were CRO^R^ (MIC > 0.125 mg/L). In total, 72.7% of ESC^R^ isolates were clonal and associated with mosaic *penA* 10 and 60 alleles. Non-mosaic *penA* 18 allele and substitutions of PenA A501T, G542S, and PorB1b G213S/Y were observed in non-clonal ESC^R^. Approximately 6.8% of the isolates showed AZM MIC above the epidemiological cutoff (ECOFF, 1 mg/L), were associated with 23S rRNA A2059G mutation, and did not exhibit clonal distribution. Almost all isolates were CIP^R^ (resistance to ciprofloxacin) and associated with GyrA-91/92 and ParC-85/86/87/88/89/91 alterations. Isolates with ParC S88P substitution were clustered into the ESC^R^ clade. The Shanghai isolates exhibited a high level of ESC^R^ and distinct resistant patterns.

## Introduction

*Neisseria gonorrhoeae* is the causative agent of gonorrhea. The World Health Organization (WHO) estimated that *N. gonorrhoeae* causes more than 86.9 million new infections worldwide annually (World Health Organization [WHO], 2018). Meanwhile, gonococcal antimicrobial resistance (AMR) continues to spread worldwide and could lead to a pandemic of extensively drug-resistant gonococci (World Health Organization [WHO], 2018). Of particular concern is the fact that ESC^RS^ [reduced susceptibility to the extended-spectrum cephalosporins (ESCs), i.e., cefixime (CFM), and ceftriaxone (CRO)], which is the first-line empirical treatment for *N. gonorrhoeae* infections (Unemo and Shafer, 2014; Wi et al., 2017), is becoming widely spread. According to the WHO Global Gonococcal Antimicrobial Surveillance Programme (GASP), in 2016, about one-third of the participating countries reported that ≥5% of isolates are resistant to ESCs (CRO and/or CFM), and half reported ≥5% resistance to azithromycin (AZM^R^). Of the 59 countries reporting ciprofloxacin resistance (CIP^R^), 95% reported ≥5% resistance and 17% reported >90% resistance (Wi et al., 2017; World Health Organization [WHO], 2018). In China, from 2013 to 2016, high prevalence of decreased susceptibility to CRO (CRO^RS^) (9.7–12.2%, MIC ≥ 0.125 mg/L) and AZM^R^ (18.6%, MIC ≥ 1.0 mg/L) has been reported (Yin et al., 2018). In Shanghai, the proportion of CRO^RS^ (MIC ≥ 0.125 mg/L) ranged from 7 to 13% during 1988–2013 (Gu et al., 2014).

Drug-resistant *N. gonorrhoeae* has been attributed to several molecular mechanisms. The primary mechanism for ESC^R^ (resistance to ESC) is mutations of the *penA* gene (encodes penicillin (PEN)-binding protein 2, PBP2, PenA), including a recombinant mosaic allele from commensal *Neisseria* (Ameyama et al., 2002; Lee et al., 2010). Mutations in the Mtr repressor genes *mtrR* and *porB* have been shown to contribute to ESC^R^ (Barry and Klausner, 2009; Unemo and Shafer, 2014). Loci involved in other AMR include mutations of 23S rRNA (Ng et al., 2002) and *mtrR* (Zarantonelli et al., 1999) for AZM^R^, mutations in *gyrA* and *parC* for CIP^R^ (Yang et al., 2006), and mutations in 16S rRNA and *rpsE* for spectinomycin (SPT) (Galimand et al., 2000; Unemo et al., 2013). Currently, the identified resistance determinants do not fully account for the observed drug resistance, and thus, other factors may be involved (Unemo and Shafer, 2014).

Genetic analysis has provided insight into outbreaks and transmission networks for several pathogens with greater resolution than traditional methods (Diep, 2013). Using genetic methods, researchers found that ESC^RS^ in Canada first emerged from a group of diverse isolates in the 1990s with non-mosaic *penA* alleles, followed in 2000/2001 with the mosaic *penA* 10 allele and then in 2007 with the mosaic *penA* 34 allele (Demczuk et al., 2015). ESC^RS^ strains in the United States are mainly clonal and associated with the mosaic *penA* 34 allele and derivatives, whereas AZM^R^ strains have arisen through multiple mechanisms and show limited clonal spread (Grad et al., 2014, 2016). To date, reported cases of CRO^R^ are sporadic, except for the FC428 strain, which was first identified in Japan in 2015 and has since then been observed in other countries (Lahra et al., 2018; Lee et al., 2019).

Genetic analysis has been used to study strain distribution along with multi-locus sequence typing (MLST) (Unemo and Dillon, 2011), *N. gonorrhoeae* multi-antigen sequence typing (NG-MAST) (Unemo and Dillon, 2011), *N. gonorrhoeae* sequence typing for AMR (NG-STAR) (Demczuk et al., 2017), and whole-genome sequencing (De Silva et al., 2016; Harris et al., 2018; Lee et al., 2018). We have reported NG-STAR analysis of seven loci in 124 *N. gonorrhoeae* isolates (Yang et al., 2020); specific NG-STAR genotypes are found to be associated with ESC^R^ and AZM^R^.

The objectives of this study were to assess whether nine loci can increase the resolution of genetic analysis and to determine the association of genetic characterization and ESC^R^ phenotypes in *N. gonorrhoeae* isolates in Shanghai. This is the first in-depth genomic analysis based on nine AMR-associated loci in *N. gonorrhoeae* in a high-level AMR setting. This study provides solid information on the molecular mechanisms and genetic characteristics of AMR in *N. gonorrhoeae* in Shanghai.

## Materials and Methods

### *Neisseria gonorrhoeae* Isolate Collection and Antimicrobial Susceptibility Testing

*Neisseria gonorrhoeae* isolates were collected from male patients with uncomplicated urogenital gonorrhea (symptoms may include pain or a burning sensation when urinating, a greater frequency or urgency of urination, and abnormal urinary discharge) at the Shanghai Skin Disease Hospital in conjunction with the China GASP. Patient consent was obtained, and ethics approval was received from the Shanghai Skin Disease Hospital. The first 30 *N. gonorrhoeae* isolates of each month in 2017 (except for 36 isolates collected in July to avoid recovery failure, making a total of 366 isolates) were used in this study. Basic demographic data of all patients were collected. The median age was 34 years (range: 18–69). Of the 366 subjects, 363 (99.2%) were ethnic Han. All patients were heterosexual. A majority of the patients had abnormal urinary discharge (98.9%). Approximately 16.4% of the patients had previous history of gonorrhea, and 12.8% received antibiotic treatment in the past month. One isolate was collected from one patient. Briefly, one urogenital specimen was collected using sterile Dacron swab and streaked on Thayer–Martin (T–M) medium (Oxoid; GuangZhou LOSO Science, Ltd.) supplemented with 1% IsoVitaleX (Oxoid; GuangZhou LOSO Science, Ltd.). *N. gonorrhoeae* was identified using criteria that included an oxidase test, Gram staining, and glucose utilization test (WHO Western Pacific Gonococccal Antimicrobial Surveillance Programme, 2008). One identified *N. gonorrhoeae* isolate for each patient was collected and stored at −70°C. Minimum inhibitory concentrations (MICs) for seven antimicrobials, including PEN, tetracycline (TET), ciprofloxacin (CIP), azithromycin (AZM), CFM, CRO, and SPT, were determined using the agar dilution method. Antimicrobial agents were purchased from Shanghai ANPEL Scientific Instrument, Co., Ltd. (Shanghai, China; distributors of Sigma-Aldrich, United States). Each MIC determination was performed in duplicate, and *N. gonorrhoeae* ATCC 49226 strain was used as a reference strain. Antimicrobial susceptibility testing results were interpreted using the EUCAST (2020) breakpoints.

### DNA Sequencing and Analysis

As previously reported, a total of 124 *N. gonorrhoeae* isolates (first 10 isolates of each month, one CIP susceptible isolate, and three isolates with CRO MICs ≥ 1.0 mg/L) were subjected to genetic analysis (Yang et al., 2020). Genomic DNA from each isolate was extracted using the Genomic DNA Purification Kit (Shanghai Promega Biological Products, Ltd., Shanghai, China). Seven loci (*penA*, *mtrR*, *porB*, *ponA*, *gyrA*, *parC*, and 23S rRNA) were PCR amplified as described previously (Yang et al., 2020). 16S rRNA was amplified by PCR (Perkin Elmer 9600 Thermocycler; Perkin Elmer, Wellesley, MA United States) using primer pair 16S-F (5’-TGATCCARCCGCASSTTC-3’) and 16S-R (5’-AGAGTTTGATCYTGGYTYAG-3’), while *rpsE* was amplified by PCR using primer pair rpsE-F (5’-TGGCAAAACATGAAATTGAAG-3’) and rpsE-R (5’-GCCATGGTTAACTCCCAAAA-3’). All primers were purchased from Invitrogen. PCR products were purified using a PCR Purification Kit (Sangon Biotech Co., Shanghai, China). DNA sequencing was performed at Sangon Biotech Co., using 3730XL (Applied Biosystems, United States) using the Sanger sequencing method. DNA sequences were verified and edited using Geneious (11.1.4)^1^ and Vector NTI Advance 11.5.3 (Lu and Moriyama, 2004). Sequences were compared with the corresponding sequences of an antimicrobial-susceptible *N. gonorrhoeae* strain FA1090. PenA amino acid sequences were compared to a wild-type PenA (Spratt, 1988) (GenBank accession number M32091). NG-STAR and NG-MAST were also performed and reported previously (Yang et al., 2020). The DNA sequences of *penA*, *mtrR*, *porB*, *ponA*, *gyrA*, *parC*, and 23S rRNA were also as previously reported (Yang et al., 2020). The DNA sequences of 16S rRNA and *rpsE* were submitted to GenBank (accession numbers MK620715–MK620729 for 16S rRNA and MN823292–MN823293 for *rpsE*).

### Phylogenetic Analysis

The sequences of all nine loci were concatenated for each strain. IQ-TREE (v1.6.12) (Nguyen et al., 2015) was used for constructing maximum-likelihood phylogenies with 1,000 bootstraps, and the best-fit model was autodetected. Phylogenies were assessed using midpoint rooting. Phylogenetic clades were determined by cluster analysis using ClusterPicker (Ragonnet-Cronin et al., 2013) with the following settings: initial and main support thresholds of 90 and genetic distance threshold of 4.5. Phylogenies and metadata (including MICs, AMR phenotypes, and molecular profiles associated with AMR) were visualized in FigTree^2^ and phandango (Hadfield et al., 2017).

### Statistical Analysis

The χ^2^ test was used to identify AMR determinants associated with CFM^R^, CRO^R^, and MICs above ECOFF for AZM using R (version 3.4.1). Multiple linear regression analysis was performed using R (version 3.4.1), to determine the relationship of log_10_ (CRO/CFM/AZM MICs) or CIP MIC intervals as the dependent variable to the presence of gene mutations.

## Results

### Antimicrobial Susceptibility of *N. gonorrhoeae* Isolates

Among the 366 *N. gonorrhoeae* isolates, 5.5% of the isolates were CRO^R^ (MICs > 0.125 mg/L), and 18.6% of isolates had CRO MICs ≥ 0.125 mg/L (Table 1 and Supplementary Table S1). About 19.4% of the 366 isolates were CFM^R^ (MICs > 0.125 mg/L). About 6.8% of the isolates showed AZM MIC above the epidemiological cutoff (ECOFF, 1 mg/L), and 99.5% of the isolates were CIP^R^. The percentages of PEN^R^ and TET^R^ were 82.5% and 60.9%, respectively. One isolate was SPT^R^. Demographic/clinical information including age, ethnicity, abnormal urinary discharge, previous history of gonorrhea, and antibiotic use in the past month was not associated with resistance to CFM, CRO, and AZM (Supplementary Table S2).

**TABLE 1:** MIC distribution of seven antimicrobial agents for 366 *N. gonorrhoeae* isolates from Shanghai.

MICs (mg/L)																		
Antimicrobials	0.002	0.004	0.008	0.016	0.03	0.06	0.125	0.25	0.5	1	2	4	8	16	32	64	≥128	≥256
**Ceftriaxone**																		
*n*^*a*^		5	11	39	135	110	46	16	1	3								
Cum%^*b*^		1.4	4.4	15	51.9	82	94.5	98.9	99.2	100								
**Cefixime**																		
*n*		2	5	22	63	125	78	37	23	7	1	3						
Cum%		0.5	1.9	7.9	25.1	59.3	80.6	90.7	97	98.9	99.2	100						
**Azithromycin**																		
*n*			1	2	14	36	101	116	63	8	4	2	9	3				7
Cum%			0.3	0.8	4.6	14.5	42.1	73.8	91	93.2	94.3	94.8	97.3	98.1				100
**Ciprofloxacin**																		
*n*	0	1	0	1	0	0	0	0	0	6	20	53	81	173	31			
Cum%	0	0.3	0.3	0.5	0.5	0.5	0.5	0.5	0.5	2.2	7.7	22.1	44.3	91.5	100			
**Penicillin**																		
*n*			0	0	0	0	2	5	4	53	95	63	17	75	52			
Cum%			0	0	0	0	0.5	1.9	3	17.5	43.4	60.7	65.3	85.8	100			
**Tetracycline**																		
*n*			0	0	0	0	2	12	27	102	107	17	1	29	69			
Cum%			0	0	0	0	0.5	3.8	11.2	39.1	68.3	73	73.2	81.1	100			
**Spectinomycin**																		
*n*											0	18	65	170	106	6	1	
Cum%											0	4.9	22.7	69.1	98.1	99.7	100	

*Vertical lines indicate the breakpoint concentrations for each antimicrobial. Breakpoints: MICs > 0.125 mg/L for ceftriaxone or cefixime, ECOFF value is 1 mg/L for azithromycin, MICs > 0.06 mg/L for ciprofloxacin MICs > 1 mg/L for penicillin or tetracycline, and MICs > 64 mg/L for spectinomycin (EUCAST, 2020). ^*a*^Number of isolates. ^*b*^Cumulative percentage.*

Supplementary Table S3 shows that 24.0% (88/366) of the sequenced isolates exhibited multidrug-resistant (MDR) phenotypes (resistance to ESC or AZM plus resistance to at least two other antimicrobials) (Martin et al., 2019). Among these phenotypes, ESC-associated phenotypes accounted for 17.7%, and AZM-associated phenotype accounted for 6.3%. Extensively drug-resistant phenotypes (resistance to ESC and AZM plus resistance to at least two other antimicrobials) (Martin et al., 2019) were noted in two isolates, namely, CFM^R^ (MIC = 0.25 mg/L)–AZM^R^ (MIC = 2 mg/L)–CIP^R^ (MIC ≥ 16 mg/L)–PEN^R^ (MIC = 4 mg/L)–TET^R^ (MIC = 4 mg/L) and CRO^R^ (MIC = 0.25 mg/L)–CFM^R^ (MIC = 0.25 mg/L)–AZM^R^ (MIC ≥ 8 mg/L)–CIP^R^ (MIC ≥ 16 mg/L)-PEN^R^ (MIC ≥ 16 mg/L)–TET^R^ (MIC = 2 mg/L).

### Genotyping of *N. gonorrhoeae* ESC^R^ Isolates

#### Mosaic *penA* and Substitutions in PenA and Association With NG-STAR Types

Approximately 76.2% of the CFM^R^ isolates (16/21) had mosaic *penA* alleles, and only 2.9% of the CFM^*S*^ isolates (3/103) possessed mosaic *penA* alleles (Table 2). Mosaic *penA* 10 and 60 alleles and substitutions in the mosaic *penA* coding region such as D101E and A549T were significantly associated with CFM^R^. Specifically, 10 out of 11 (90.9%) *penA*-10.001 isolates were CFM^R^, and four out of four (100%) *penA*-60.001 isolates were CFM^R^. Substitutions of F374V, H541N, P552V, KI555QV, I566V, and A574V were also statistically associated with CFM^R^.

**TABLE 2:** Molecular profiles associated with resistance to cefixime, ceftriaxone, and azithromycin in *N. gonorrhoeae*.

	All isolates						Non-mosaic *penA* isolates						All isolates		

Molecular markers	CFM^*S*^ *n* (%)	CFM^R^ *n* (%)	*p*	CRO^*S*^ *n* (%)	CRO^R^ *n* (%)	*p*	CFM^*S*^ *n* (%)	CFM^R^ *n* (%)	*p*	CRO^*S*^ *n* (%)	CRO^R^ *n* (%)	*p*	<AZM ECOFF *n* (%)	>AZM ECOFF *n* (%)	*p*
**NG-STAR genotype^*a*^ (*penA* type)**															
ST-202 (*penA* 2)	4 (3.9)	0 (0)	1	4 (3.4)	0 (0)	1							**0 (0)**	**4 (50.0)**	**<0.0001**
ST-233 (*penA* 60)	**0 (0)**	**3 (14.3)**	**0.004**	**0 (0)**	**3 (37.5)**	**<0.001**							3 (2.6)	0 (0)	1
ST-348 (*penA* 10)	**1 (1.0)**	**4 (19.0)**	**0.003**	5 (4.3)	0 (0)	1							5 (4.3)	0 (0)	1
ST-428 (*penA* 18)	3 (2.9)	2 (9.5)	0.199	**3 (2.6)**	**2 (25.0)**	**0.033**	**3 (3.0)**	**2 (40.0)**	**0.017**	**3 (3.0)**	**2 (40.0)**	**0.017**	5 (4.3)	0 (0)	1
***penA* type**															
Mosaic	**3 (2.9)**	**16 (76.2)**	**<0.0001**	16 (13.8)	3 (37.5)	0.196									
*penA* 10	**1 (1.0)**	**10 (47.6)**	**<0.0001**	11 (9.5)	0 (0)	1									
*penA* 34	2 (1.9)	1 (4.8)	0.430	3 (2.6)	0 (0)	1									
*penA* 60	**0 (0)**	**4 (19.0)**	**<0.001**	**1 (0.9)**	**3 (37.5)**	**<0.001**									
*penA* 71	0 (0)	1 (4.8)	0.169	1 (0.9)	0 (0)	1									
Non-mosaic	**100 (97.1)**	**5 (23.8)**	**<0.0001**	100 (86.2)	5 (62.5)	0.196									
*penA* 18	15 (14.6)	3 (14.3)	0.759	**14 (12.1)**	**4 (50.0)**	**0.015**	**15 (15.0)**	**3 (60.0)**	**0.034**	**14 (14.0)**	**4 (80.0)**	**0.003**			
**Substitutions in mosaic *penA***															
D101E plus^*b*^	**3 (2.9)**	**11 (52.4)**	**<0.0001**	14 (12.1)	0 (0)	0.599									
YGED201HAGE, Q214E	**4 (3.9)**	**12 (57.1)**	**<0.0001**	16 (13.8)	0 (0)	0.562									
A311V, T483S, T485I	**0 (0)**	**4 (19.0)**	**<0.001**	**1 (0.9)**	**3 (37.5)**	**<0.001**									
I312M, V316T, N512Y plus^*c*^	**3 (2.9)**	**16 (76.2)**	**<0.0001**	16 (13.8)	3 (37.5)	0.196									
P341S	**3 (2.9)**	**12 (57.1)**	**<0.0001**	15 (12.9)	0 (0)	0.594									
GA375TP	**3 (2.9)**	**13 (61.9)**	**<0.0001**	16 (13.8)	0 (0)	0.562									
A549T	**1 (1.0)**	**15 (71.4)**	**<0.0001**	13 (11.2)	3 (37.5)	0.061									
**PenA Substitutions**															
F374V	**0 (0)**	**4 (19.0)**	**<0.001**	**1 (0.9)**	**3 (37.5)**	**<0.001**									
A501T^*d*^	15 (14.6)	3 (14.3)	0.759	**14 (12.1)**	**4 (50.0)**	**0.015**	**15 (15.0)**	**3 (60.0)**	**0.034**	**14 (14.0)**	**4 (80.0)**	**0.003**			
A501V	**58 (56.3)**	**1 (4.8)**	**<0.0001**	58 (50.0)	1 (12.5)	0.091									
A516G	**100 (97.1)**	**5 (23.8)**	**<0.0001**	100 (86.2)	5 (62.5)	0.119									
H541N	**23 (22.3)**	**16 (76.2)**	**<0.0001**	36 (31.0)	3 (37.5)	0.990									
G542S	27 (26.2)	3 (14.3)	0.245	26 (22.4)	4 (50.0)	0.182	27^*e*^ (27.0)	3^*f*^ (60.0)	0.277	**26**^*g*^ **(26.0)**	**4**^*h*^ **(80.0)**	**0.036**			
P552V, KI555QV	**17 (16.5)**	**15 (71.4)**	**<0.0001**	29 (25.0)	3 (37.5)	0.716									
I566V, A574NV	**47 (45.6)**	**18 (85.7)**	**<0.001**	58 (50)	7 (87.5)	0.091									
***porB* genotype**															
*porB*1*a*	7 (6.8)	1 (4.8)	1	8 (6.9)	0 (0)	1									
*porB*1*b*	96 (93.2)	20 (95.2)	1	108 (93.1)	8 (100)	1									
**PorB1b Substitutions**															
T87A	8 (8.3)	2 (10.0)	0.844	**7 (6.5)**	**3 (37.5)**	**0.021**									
T89S	10 (10.4)	3 (15.0)	0.816	**10 (9.3)**	**3 (37.5)**	**0.045**									
GA120KD	52 (54.2)	15 (75.0)	0.086	60 (55.6)	7 (87.5)	0.163									
G213S/Y	11 (11.5)	4 (20.0)	0.503	**10 (9.3)**	**5 (62.5)**	**<0.001**	11 (11.8)	2 (40.0)	0.1294	**10 (10.8)**	**3 (60.0)**	**0.016**			
Q214L	7 (7.3)	2 (10.0)	0.962	**6 (5.6)**	**3 (37.5)**	**0.015**									
G259A	61 (63.5)	17 (85.0)	0.063	**70 (64.8)**	**8 (100)**	**0.041**									
**PonA Substitutions**															
T375A	100 (97.1)	21 (100)	1	113 (97.4)	8 (100)	1									
L421P	99 (96.1)	21 (100)	1	112 (96.6)	8 (100)	1									
**MtrR Substitutions**															
A39T	10 (9.7)	3 (14.3)	0.816	13 (11.2)	0 (0)	1							13 (11.20)	0 (0)	1
A40D	**4 (3.9)**	**8 (38.1)**	**<0.0001**	12 (10.3)	0 (0)	1							12 (10.3)	0 (0)	1
G45D	**33 (32)**	**1 (4.8)**	**0.011**	32 (27.6)	2 (25)	1							**28 (24.1)**	**6 (75.0)**	**0.007**
T86A	**10 (9.7)**	**8 (38.1)**	**0.002**	18 (15.5)	0 (0)	0.493							18 (15.5)	0 (0)	0.493
H105Y	49 (47.6)	8 (38.1)	0.427	51 (44)	6 (75)	0.181							56 (48.3)	1 (12.5)	0.110
promoter -35A	**88 (85.4)**	**10 (47.6)**	**<0.001**	91 (78.4)	7 (87.5)	0.873							90 (77.6)	8 (100)	0.290
**23S rRNA Mutations**															
A2059G													**0 (0)**	**7 (87.5)**	**<0.0001**

*χ^2^ test was used to identify molecular profiles associated with resistance to cefixime (CFM^R^), ceftriaxone (CRO^R^), and azithromycin (>AZM ECOFF). ^*a*^NG-STAR genotypes were reported previously (Yang et al., 2020). ^*b*^Also including substitutions V160A, N173S, A280V, D285E, R288K, and R291Q. ^*c*^Also including substitutions A323S, T326V, L328A/P, NERL329TDTF, Q335L, SP342AT, RD345Q, S352T, R373M, E377K, E385D, I388V, N406S, RP411QK, A437V, V443E, L447V, IF461VI, E464A, Q457K, RE468KK, N472E, P480A, and G545S. ^*d,f,h*^All isolates were *penA* 18 allele. ^*e*^Including 10 isolates with *penA* 5, 1 isolate with *penA* 17, 15 isolates with *penA* 18, and 1 isolate with *penA* 41. ^*g*^Including 10 isolates with *penA* 5, 1 isolate with *penA* 17, 14 isolates with *penA* 18, and 1 isolate with *penA* 41. Bold values indicate *p* < 0.05.*

Mosaic *penA* alleles were detected in 37.5% of CRO^R^ isolates and in 13.8% of CRO^*S*^ isolates. Only the mosaic *penA* 60 allele was significantly associated with CRO^R^ (Table 2). PenA substitutions F374V and A501T showed significantly higher frequencies in CRO^R^ isolates than in CRO^*S*^ isolates.

NG-STAR ST-233 (*penA* 60) was associated with CFM^R^ and CRO^R^ (Table 2), while ST-348 (*penA* 10, exhibited by NG-MAST ST5308, ST7554, and ST12784) was associated with CFM^R^ and ST-428 (*penA* 18) was associated with CRO^R^.

The metadata of four mosaic *penA* 60 isolates are listed in Table 3. Demographic and clinical information revealed that four patients were young (age range: 18–44), all of them had abnormal urinary discharge, and none reported previous history of gonorrhea or any antibiotic use in the past month. Three of four mosaic *penA* 60 isolates were NG-STAR genotype 233, whereas one was NG-STAR genotype 1143. Four *penA* 60 isolates had the same pattern of PenA substitutions, which contained A311V and T483S alterations, the key CRO^R^ substitution. All *penA 60* isolates have identical *ponA*, *mtrR*, 23S rRNA, *gyrA*, *parC*, 16S rRNA, and *rpsE* patterns and different PorB substitutions. Additional MICs and the molecular profiles of four isolates are summarized in Table 3.

**TABLE 3:** Metadata of four mosaic *penA* 60.001 isolates in Shanghai.

	Demographic/clinical information									MICs (mg/L)						

Isolate id	Age	Gender	Ethnicity	Transmission	Infection site	Abnormal urinary discharge	Previous history of gonorrhea	Antibiotic use in the past month	Date of clinic visit	CRO	CFM	AZM	CIP	PEN	TET	SPT
17–256	44	Male	Han	Hetero	Urethra	Yes	No	No	02/12/2017	0.125	0.5	0.06	16	≥32	2	8
SH-40	18	Male	Han	Hetero	Urethra	Yes	No	No	25/11/2017	1	≥4	0.125	≥32	4	2	16
SH-41	35	Male	Han	Hetero	Urethra	Yes	No	No	27/11/2017	1	≥4	0.125	≥32	2	1	8
SH-48	28	Male	Han	Hetero	Urethra	Yes	No	No	06/12/2017	1	≥4	0.125	≥32	4	2	8

	**Molecular profiles**															

**Isolate ID**	**NG-STAR genotype**	**PPNG**	***penA***	**PorB1b**	**PonA**	**MtrR**	**23S rRNA**	**GyrA**	**ParC**	**16S rRNA**	***rpsE***					

17–256	1143	PPNG	60.001	G120K, A121G, Q143K, T215A, I218M, M257R, S258R, G259A	T375A, L421P	Promoter −35A, H105Y	WT	S91F, D95A	S87R	T1458C	WT					
SH-40	233	Non-PPNG	60.001	T87A, T89S, G120K, A121D, V151A, I209M, YD211GY, G213Y, Q214L, T215, S217N, V242A, A256T, M257S, G259A, A272V	T375A, L421P	Promoter −35A, H105Y	WT	S91F, D95A	S87R	T1458C	WT					
SH-41	233	Non-PPNG	60.001	G120K, A121D, Q143K, T215V, M257R, S258R, G259A	T375A, L421P	Promoter −35A, H105Y	WT	S91F, D95A	S87R	T1458C	WT					
SH-48	233	Non-PPNG	60.001	T87A, T89S, G120K, A121D, V151A, I209M, YD211GY, G213Y, Q214L, T215, S217N, V242A, A256T, M257S, G259A, A272V	T375A, L421P	Promoter −35A, H105Y	WT	S91F, D95A	S87R	T1458C	WT					
																

Among the non-mosaic *penA* allele isolates, the *penA* 18 allele and substitutions of PenA A501T and G542S were associated with ESC^R^ (Table 2). The proportion of non-mosaic *penA* ESC^R^ isolates harboring the PenA A501T substitution (for CFM, 60%, 3/5; for CRO, 80%, 4/5) was significantly higher than the proportion of non-mosaic *penA* ESC^*S*^ isolates harboring that substitution (for CFM, 15%, 15/100; for CRO, 14%, 14/100). Eighty percent (4/5) of non-mosaic *penA* CRO^R^ isolates had the G542S substitution, which was significantly higher than non-mosaic *penA* CRO^*S*^ isolates (26%, 26/100). Interestingly, all ESC^R^ with the PenA double substitutions of A501T and G542S (three CFM^R^ and four CRO^R^) exhibited a *penA* 18 allele.

### *porB1a* and *porB1b* Genes

Among the 124 isolates, genotypes *porB1a* and *porB1b* accounted for 6.5% and 93.5%, respectively. CRO^R^ isolates had a higher percentage of PorB1b substitutions T87A, T89S, G213S/Y, Q214L, and G259A than CRO^*S*^ isolates (Table 2).

Among non-mosaic *penA* allele isolates, 60% (3/5) of CRO^R^ non-mosaic *penA* isolates harbored the PorB1b G213S/Y substitution, which was significantly higher than the CRO^*S*^ non-mosaic *penA* isolates (10.8%, 10/93) (Table 2).

### *mtrR* Gene and Promoter

MtrR G45D and *mtrR* promoter -35A were significantly lower in CFM^R^ isolates than in CFM^*S*^ isolates. MtrR A40D and T86A were significantly associated with CFM^R^. No *mtrR* mutations was found to be associated with CRO^R^.

### Characteristic Genotypes of *N. gonorrhoeae* Isolates With MICs Above AZM ECOFF

In isolates with MICs above the AZM ECOFF value (1 mg/L), 87.5% (7/8, AZM MICs ≥ 256 mg/L) harbored the A2059G mutation in 23S rRNA, which is significantly higher than in isolates with MICs below AZM ECOFF (0/116, AZM MIC range: ≤0.03–1 mg/L). MtrR G45D and NG-STAR ST-202 (NG-MAST ST1866) were significantly higher in isolates above AZM ECOFF than in isolates below AZM ECOFF (*p* = 0.007 for MtrR G45D, *p* < 0.0001 for NG-STAR ST-202).

### Characteristic Genotypes in *N. gonorrhoeae* SPT^R^ Isolates

**FIGURE 1:**
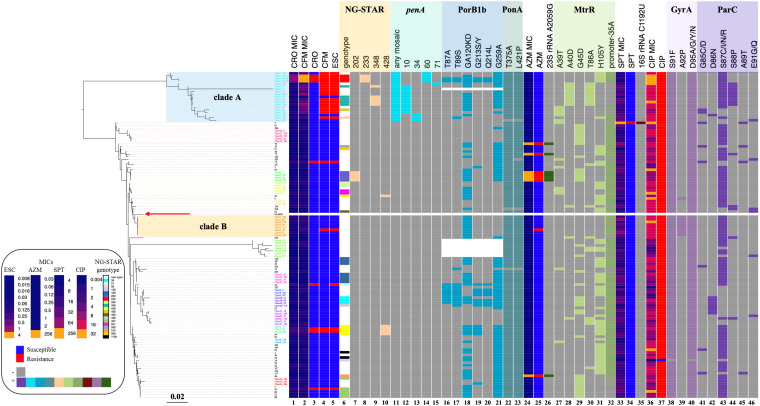
Phylogenetic reconstruction of nine genes, patterns of antimicrobial resistance, and genetic polymorphisms in 124 *N. gonorrhoeae* isolates. Left: Phylogenetic reconstruction of 124 isolates based on maximum likelihood. The red arrow indicates the reference strain FA1090. Heatmap. Columns 1 and 2: MIC values of CRO and CFM. Columns 3–5: Susceptible/resistant categories according to the EUCAST MIC breakpoints of CRO, CFM, and ESCs. Column 6: NG-STAR genotype (white band indicates NG-STAR genotypes new to the NG-STAR database). Columns 7–10: A specific NG-STAR genotype. Column 11: Any mosaic *penA* allele. Columns 12–15: A specific mosaic *penA* allele. Columns 16–21: Non-synonymous amino acid changes from wild type in PorB1b. Columns 22 and 23: Non-synonymous amino acid changes from wild type in PonA. Column 24: MIC values of AZM. Column 25: Susceptible/resistant categories according to the EUCAST MIC breakpoints of AZM. Column 26: A2059G mutation in 23S rRNA. Columns 27–31: Non-synonymous amino acid changes from wild type in MtrR. Column 32: The –35A deletion in the *mtrR* promoter. Column 33: MIC values of SPT. Column 34: Susceptible/resistant categories according to the EUCAST MIC breakpoints of SPT. Column 35: C1192U mutation in 16S rRNA. Column 36: MIC values of CIP. Column 37: Susceptible/resistant categories according to the EUCAST MIC breakpoint of CIP. Columns 38–40: Non-synonymous amino acid changes from wild type in GyrA. Columns 41–46: Non-synonymous amino acid changes from wild type in ParC. The purple and orange rectangles indicate clades A and B described in the text, respectively. Sequence names are colored by cluster.

There was only one SPT^R^ isolate identified. This SPT^R^ isolate was the only strain that harbored 16S rRNA C1192U mutation in our dataset (Figure 1), which has earlier been reported to be associated with SPT^R^. The K26E substitution in RpsE was not detected.

### Clonal Distribution of *N. gonorrhoeae* ESC^R^ Isolates by Phylogenetic Analysis

Phylogenetic analysis of nine genes was performed. Compared to a seven-gene phylogeny (Supplementary Figure 1), the inclusion of two SPT^R^ genes (16S rRNA and rpsE) did not change the structure or resolution of the phylogeny. Cluster analysis results showed that a nine-gene phylogeny had 14 clusters, while a seven-gene phylogeny had 16 clusters. Most ESC^R^ strains were classified as one clade that had the mosaic *penA* alleles (Figure 1, clade A). NG-STAR ST-233, ST-348, and ST-90 belonged to clade A. Approximately 76% (16 of 21) of CFM^R^ were included in clade A (Figure 1). CRO^R^ appeared sporadically across the phylogeny.

**FIGURE 2:**
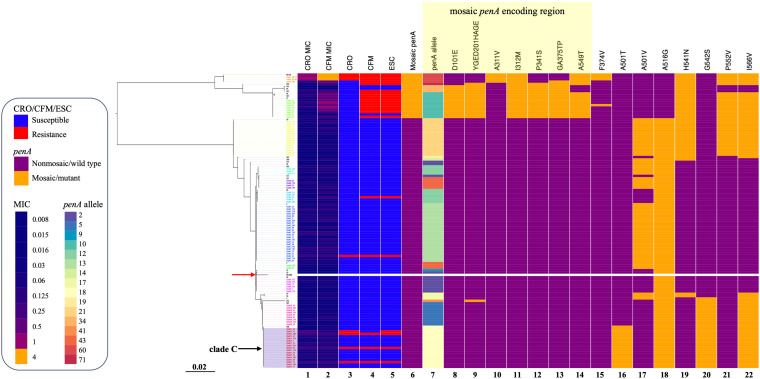
*penA* gene phylogeny, patterns of antimicrobial resistance, and genetic polymorphisms of 124 *N. gonorrhoeae* isolates. Left: Phylogenetic reconstruction of 124 isolates based on the *penA* gene and using the maximum likelihood. The red arrow indicates reference strain FA1090. Heatmap. Columns 1 and 2: MIC values of CRO and CFM. Columns 3–5: Susceptible/resistant categories according to the EUCAST MIC breakpoints of CRO, CFM, and ESCs. Column 6: Any mosaic *penA* allele. Column 7: *penA* alleles. Columns 8–22: Non-synonymous amino acid changes from wild type in PenA. The purple rectangle indicates clade C as described in the text. The yellow rectangle indicates substitutions in the mosaic *penA* coding region. Sequence names are colored by cluster.

Four of the six ESC^R^ that did not possess a mosaic *penA* allele harbored a *penA* 18 allele and was classified into clade C (Figure 2). The *penA* 18 allele consisted of the A501T and G542S double substitutions.

Multiple linear regression analysis revealed that the mosaic *penA*, PenA A501T/V, and PorB1b G213S/Y substitutions were strongly associated with increased MICs of CRO or CFM (Supplementary Table S4).

### Phylogenetic Analysis of *N. gonorrhoeae* Isolates With MICs Above AZM ECOFF

Isolates with MICs above AZM ECOFF appeared sporadically across the phylogenetic tree (Figure 1) and were highly associated with the 23S rRNA A2059G mutation (Table 2 and Figure 1). All NG-STAR ST-202 isolates harbored the 23S rRNA A2059G mutation. Multiple linear regression analysis indicated that the 23S rRNA A2059G mutation was strongly associated with increased AZM MICs (Supplementary Table S5).

### Analysis of *N. gonorrhoeae* CIP^R^ Isolates

**FIGURE 3:**
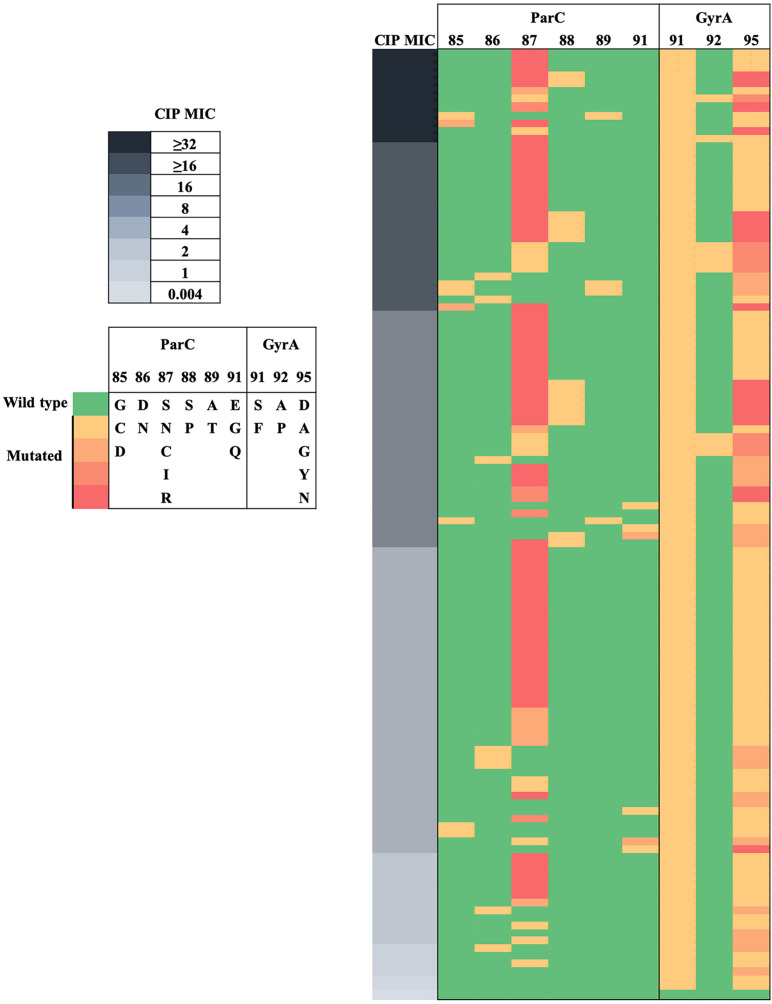
Heat map visualization of CIP MICs with ParC and GyrA substitutions in *N. gonorrhoeae*. MICs of CIP are indicated on the left panel. ParC and GyrA substitutions are indicated on the middle and right panels, respectively.

The 124 isolates included 123 CIP^R^ and 1 CIP^*S*^. Substitutions at GyrA-91 and GyrA-95 were highly predictive of the resistant phenotype (Figure 3). CIP^*S*^ did not harbor GyrA or ParC substitutions. CIP^R^ with CIP MICs > 0.06 mg/L had both GyrA-91 (S91F) and GyrA-95 (D95A/G/Y/N) substitutions. *N. gonorrhoeae* isolates with triple GyrA substitutions at positions 91, 92, and 95 exhibited a high level of quinolone resistance (CIP MICs ≥ 16 mg/L). Phylogenetic analysis (Figure 1) revealed that GyrA A92P and ParC S88P substitutions could be clustered into two clades, whereas other GyrA and ParC substitutions were distributed across the phylogeny. Specifically, eight out of nine (88.9%) isolates with GyrA A92P substitution were clustered into clade B, whereas 9 out of 14 (64.3%) isolates with ParC S88P substitution were clustered into clade A. Multiple linear regression analysis indicated that ParC-85/86/87/88/89/91 and GyrA-91/92 substitutions heavily contributed to CIP MIC increments (Supplementary Table S6).

## Discussion

A large proportion of *N. gonorrhoeae* isolates in Shanghai in 2017 exhibited resistance to ESCs. Approximately 19.4% of 366 *N. gonorrhoeae* isolates were CFM^R^ (MICs > 0.125 mg/L), and 40.7% of the isolates had CFM MICs ≥ 0.125 mg/L. About 5.5% of the isolates were CRO^R^ (MICs > 0.125 mg/L), and 18.0% of isolates had CRO MICs ≥ 0.125 mg/L. About 6.8% of the isolates had MICs above AZM ECOFF. One isolate was SPT^R^. *N. gonorrhoeae* CFM^R^ isolates exhibited clonal distribution of one cluster containing mosaic *penA* alleles, whereas CRO^R^ isolates appeared sporadically across the phylogeny.

The resistant percentages of *N. gonorrhoeae* isolates to CRO, CFM, or AZM in Shanghai exceeded the WHO cutoff of 5%, indicating a need to review recommended treatments (World Health Organization [WHO], 2012). Over 18.0% of *N. gonorrhoeae* isolates had CRO MICs ≥ 0.125 mg/L, higher than that reported in previous years in Shanghai and other places in China (Gu et al., 2014; Yin et al., 2018) as well as several other countries such as 0.1% in the United States in 2013 and 2014 (Kirkcaldy et al., 2016), 1.8% in Canada in 2016 (Martin et al., 2019), and 10.7% in Japan in 2012–2013 (Hamasuna et al., 2015). The proportion of *N. gonorrhoeae* CFM^R^ isolates (MICs > 0.125 mg/L, 19.4%) in Shanghai in 2017 was much higher than that in the United States (0.4–0.8% in 2013–2014) (Kirkcaldy et al., 2016), Europe (1.7–2.0% in 2014–2015) (Cole et al., 2017), and Canada (0.3% in 2016) (Martin et al., 2019). These findings indicate that unlike those in the United States, European countries, Japan, and Canada, CRO and CFM may need to be reviewed as a treatment for gonorrhea in Shanghai. *N. gonorrhoeae* isolates in Shanghai remain susceptible to SPT (Yang et al., 2006), suggesting that SPT may have potential as a first-line therapy for the treatment of uncomplicated urogenital gonorrhea in Shanghai. SPT is available in China. However, SPT is not suitable for the treatment of pharyngeal gonorrhea, as its efficacy rate is approximately 80% (Moran and Levine, 1995). Furthermore, SPT^R^ isolates have been reported in several countries such as the Netherlands, the Philippines, South Korea, and the United Kingdom (Unemo and Shafer, 2014). There is concern that drug resistance would be rapidly selected when SPT is introduced as a first-line monotherapy. Therefore, SPT should be considered as a first-line treatment in combination with CRO or AZM in Shanghai.

### *Neisseria gonorrhoeae* ESC^R^ Isolates Tend to Be Clonal

Previous studies have shown that *N. gonorrhoeae* CFM^R^ isolates in Japan (Yahara et al., 2018) and ESC^RS^ isolates in the United States (Grad et al., 2014, 2016), Europe (Chisholm et al., 2013), and Canada (Demczuk et al., 2015) are predominantly clonal and associated with the mosaic *penA* allele. We also found that *N. gonorrhoeae* ESC^R^ isolates were predominantly clonal in this study. In addition, we observed that ESC^R^ isolates without the *penA* mosaic alleles were distributed sporadically across the phylogenetic tree, which is also concordant with a previous report (Grad et al., 2016). In our study, of the six ESC^R^ isolates that did not possess the mosaic *penA* allele, four contained the *penA* 18 allele that included the PenA A501T and G542S double substitutions. However, ESC^RS^ lineages in Canada were associated with non-mosaic *penA* 12 and 13 alleles (Demczuk et al., 2015), while ESC^RS^ isolates with non-mosaic *penA* reported in the United States have sporadically emerged even in the *penA* gene phylogeny (Grad et al., 2016). The sample size of *N. gonorrhoeae* isolates in this study could be expanded, and whole-genome sequencing should be examined to further confirm this difference.

### Mosaic *penA* Alleles Are Associated With *N. gonorrhoeae* ESC^R^ and NG-STAR Clusters

Mosaic *penA* alleles have been associated with *N. gonorrhoeae* ESC^RS^ (Ameyama et al., 2002; Lee et al., 2010). We observed that ESC^R^ is highly associated with mosaic *penA* 10, whereas reports in the United States and Canada indicated that *N. gonorrhoeae* ESC^RS^ is highly associated with mosaic *penA* 34 (Grad et al., 2014, 2016; Demczuk et al., 2015). All of the *N. gonorrhoeae* isolates with an NG-STAR ST-348 genotype (*n* = 5) contained the mosaic *penA* 10 allele. Several mosaic *penA* alleles (*penA* 60, *penA* 71, and *penA* 34) are associated with ESC^R^ in this study.

*penA* 60 is significantly associated with both CRO^R^ and CFM^R^ and occurs in a single cluster; thus, it is of great concern when it spreads. None of the carriers of the *penA* 60 isolates reported a previous history of gonorrhea or any antibiotic use in the past month, which indicates that they were recently infected with *penA* 60 ESC^R^ strains. It is important to monitor the clonal expansion of *penA* 60 ESC^R^ strains to contain its spread. The reported CRO-resistant cluster FC428 has a mosaic *penA* 60 genotype with a NG-STAR sequence type ST-233 (Lee et al., 2019). Three of the four *penA* 60 *N. gonorrhoeae* isolates also have an NG-STAR ST-233. Links between the *penA* 60 isolates in this study and FC428 strains remain to be elucidated.

### Novel PorB1b Substitutions Associated With *N. gonorrhoeae* ESC^R^

In this study, we found that in contrast to CFM^R^, CRO^R^ is apparently associated with PorB1b substitutions other than mosaic *penA* or PenA substitutions. This is concordant with the results of a previous study that CRO is more severely affected by PorB1b than CFM (Unemo and Shafer, 2014), suggesting that either CFM does not readily diffuse into the periplasm through PorB1b or such diffusion is not altered by the *porB* determinant (Unemo and Shafer, 2014). To our knowledge, our study is the first to report that PorB1b substitutions T87A, T89S, S213S/Y, Q214L, and G259A are associated with CRO^R^. Similar to a previous report, although certain mutations in *porB* can contribute to *N. gonorrhoeae* resistance, most mutations in *porB* do not (Goire et al., 2014). *In vitro* selection by introducing *porB* mutations into CRO^*S*^ isolates should be considered in the future to confirm the role of these mutations in the formation of CRO^R^ (Johnson et al., 2014). Previous studies have reported substitutions at amino acid positions 120 and 121 in putative loop 3 of PorB1b, which reduce the permeability of ESCs (Olesky et al., 2002). Interestingly, none of the substitutions detected in the present study are situated in any loops of PorB1b, and whether these substitutions could perturb protein structure remains unknown. Electrophysiological and biochemical studies of PorB1b proteins to reveal the mechanism of CRO^R^ conferred by these substitutions are warranted.

### ParC S88P Substitution Clustered in ESC^R^ Clade

We noticed that among the 14 isolates with ParC S88P substitution, nine were clustered in the ESC^R^ clade (clade A), suggesting that this substitution may be associated with ESC^R^. Intriguingly, it was reported that various MDR bacteria such as methicillin-resistant *Staphylococcus aureus* (MRSA), extended-spectrum β-lactamase (ESBL)-producing *Klebsiella pneumoniae*, and ESBL-producing *Escherichia coli* were demonstrated to have been selected by favorable fitness balance associated with high-level resistance to fluoroquinolones, principally attained by the mutations of some serine residues in *gyrA* and *parC/grlA* (Fuzi et al., 2017, 2020). The association of the ParC S88P substitution and that of other QRDR serine replacements with fitness gain, the promotion of particular clades, and the acquisition of the MDR phenotype warrant further investigation.

### Limitations

This study only investigated a small percentage of *N. gonorrhoeae* isolates in Shanghai, with a total of 5,711 reported cases in this city in 2017. However, it is representative of the institution where the isolates were collected. A study with a larger sample size is required to extrapolate a broader strain distribution and to provide convincing evidence for the clonal distribution of ESC^R^ with mosaic *penA* alleles and the sporadic distribution of ESC^R^ with non-mosaic *penA* alleles. Specimens were obtained only from male patients, which may cause a higher proportion of CRO-resistant isolates, as reported by a Chinese national surveillance (Yin et al., 2018). Transmission of gonorrhea via different behaviors may result in infection of other mucosal sites, and isolates from other sites may exhibit different AMR phenotypes and genotypes. In the future, whole-genome sequencing should be considered to examine the population structure in Shanghai *N. gonorrhoeae* isolates, which would provide a significantly higher resolution for phylogenetic reconstruction.

## Conclusion

This study observed a high percentage of *N. gonorrhoeae* isolates with reduced susceptibility to ESCs in Shanghai in 2017. Phylogenetic analysis of resistance determinants revealed that CFM^R^ isolates tend to be clonal. Mosaic *penA* alleles and certain substitutions in PenA and PorB1b are associated with *N. gonorrhoeae* ESC^R^. CRO and CFM may need to be reviewed as treatment for gonorrhea in Shanghai. Monitoring clonal expansion and development of novel antimicrobials for gonorrhea treatment are urgently needed.

## Data Availability Statement

The sequence data generated for this study has been submitted to GenBank and accession numbers can be found in the article.

## Ethics Statement

The studies involving human participants were reviewed and approved by Shanghai Skin Disease Hospital. The patients/participants provided their written informed consent to participate in this study.

## Author Contributions

YD analyzed the data and wrote the manuscript. YY performed the experiments and collected the data. YW, IM, and WD revised the manuscript. WG designed experiments, performed the experiments, collected the data, and revised the manuscript. All authors contributed to the article and approved the submitted version.

## Conflict of Interest

The authors declare that the research was conducted in the absence of any commercial or financial relationships that could be construed as a potential conflict of interest.

## Footnotes

1 https://www.geneious.com

2 http://tree.bio.ed.ac.uk/software/figtree/
