# Supplementary material for: Shanghai Neisseria gonorrhoeae Isolates Exhibit Resistance to Extended-Spectrum Cephalosporins and Clonal Distribution
Source: Front Microbiol. 2020 Oct 6;11:580399. doi: 10.3389/fmicb.2020.580399 (PMC7573285; doi:10.3389/fmicb.2020.580399)
Supplement: Supplementary file 1 [file Data_Sheet_1.docx]

**Supplementary Table S1. Antimicrobial susceptibility of *N. gonorrhoeae* isolates in Shanghai in 2017**

|  | **N=366** | | | |  | **N=124** | | | |
| --- | --- | --- | --- | --- | --- | --- | --- | --- | --- |
| **Antimicrobial** | **MIC range(mg/L)** | **S (n,%)** | **I (n,%)** | **R (n,%)** |  | **MIC range(mg/L)** | **S (n,%)** | **I (n,%)** | **R (n,%)** |
| Ceftriaxone^a^ | ≤0.004-1 | 346 (94.5) |  | 20 (5.5) |  | ≤0.008-1 | 116 (93.5) |  | 8(6.5) |
| Cefixime^b^ | ≤0.004-≥4 | 295 (80.6) |  | 71 (19.4) |  | 0.008-≥4 | 103 (83.1) |  | 21(16.9) |
| Azithromycin^c^ | ≤0.008-≥256 | 341 (93.2) |  | 25(6.8) |  | ≤0.03-≥256 | 116 (93.5) |  | 8 (6.5) |
| Ciprofloxacin^d^ | 0.004-≥32 | 2 (0.5) |  | 364 (99.5) |  | 0.004-≥32 | 1 (0.8) |  | 123 (99.2) |
| Penicillin^e^ | 0.125-≥32 |  | 64 (17.5) | 302 (82.5) |  | 0.25-≥32 |  | 23 (18.5) | 101 (81.5) |
| Tetracycline^f^ | 0.125-≥32 | 41 (11.2) | 102 (27.9) | 223 (60.9) |  | 0.125-≥32 | 13 (10.5) | 37 (29.8) | 74 (59.7) |
| Spectinomycin^g^ | ≤4-≥256 | 365 (99.7) |  | 1 (0.3) |  | 4-≥256 | 123 (99.2) |  | 1 (0.8) |

^ab^MIC≤0.125mg/L as susceptible (S), MIC>0.125mg/L as reduced susceptible (R).

^c^ ECOFF = 1 mg/L.

^d^ MIC≤0.03mg/L as susceptible (S), MIC>0.06mg/L as resistant (R).

^e^ MIC≤0.06mg/L as susceptible (S), MIC>1mg/L as resistant (R), I (Suspectible, increased exposure) is interpreted as values between the S and the R breakpoints.

^f^ MIC≤0.5mg/L as susceptible (S), MIC>1mg/L as resistant (R) , I (Suspectible, increased exposure) is interpreted as values between the S and the R breakpoints.

^g^ MIC≤64mg/L as susceptible (S), MIC>64mg/L as resistant (R).

**Supplementary Table S2. Demographic/clinical information associated with resistance to cefixime, ceftriaxone and azithromycin in *N.gonorrhoeae***

|  | **CFM^S^ n(%)** | **CFM^R^ n(%)** | **p-value** | **CRO^S^ n(%)** | **CRO^R^ n(%)** | **p-value** | **AZM^S^ n(%)** | **AZM^R^ n(%)** | **p-value** |
| --- | --- | --- | --- | --- | --- | --- | --- | --- | --- |
| Total | 295 | 71 |  | 346 | 20 |  | 341 | 25 |  |
| Age<35 | 158(53.6) | 38(53.5) | 0.995 | 184(53.2) | 12(60.0) | 0.552 | 183(53.7) | 13(52.0) | 0.872 |
| Ethic Han | 293 (99.3) | 70(98.6) | 0.477 | 344(99.4) | 19(95.0) | 0.156 | 338(99.1) | 25(100) | 1 |
| Abnomal uninary discharge | 292(99.0) | 70(98.6) | 0.580 | 342(98.8) | 20(100) | 1 | 337(98.8) | 25(100) | 1 |
| Previous history of gonorrhea | 45(15.3) | 15(21.1) | 0.230 | 58(16.8) | 2(10.0) | 0.629 | 57(16.7) | 3(12.0) | 0.738 |
| Antibiotic use in the past month | 34(11.5) | 13(18.3) | 0.125 | 43(12.4) | 4(20.0) | 0.522 | 45(13.2) | 2(8.0) | 0.660 |

**Supplementary Table S3. Multidrug-resistant and extensively drug-resistant phynotypes in 366 *N. gonorrhoeae* isolates in Shanghai, 2017**

| **Pattern** | n | **%** | **Phenotypes** | n | **%** |
| --- | --- | --- | --- | --- | --- |
| Multidrug-resistant | 88 | 24.0 | CFM-CIP-TET | 2 | 0.5 |
|  |  |  | CFM-CIP-PEN | 23 | 6.3 |
|  |  |  | CFM-CIP-PEN-TET | 23 | 6.3 |
|  |  |  | CRO-CIP-PEN-TET | 2 | 0.5 |
|  |  |  | CRO-CFM-CIP-PEN | 5 | 1.4 |
|  |  |  | CRO-CFM-CIP-PEN-TET | 10 | 2.7 |
|  |  |  | AZM-CIP-TET | 2 | 0.5 |
|  |  |  | AZM-CIP-PEN | 5 | 1.4 |
|  |  |  | AZM-CIP-PEN-TET | 16 | 4.4 |
| Extensively drug-resistant | 2 | 0.5 | CFM-AZM-CIP-PEN-TET | 1 | 0.3 |
|  |  |  | CRO-CFM-AZM-CIP-PEN-TET | 1 | 0.3 |

**Supplementary Table S4. Multiple regression analysis of the correlation of log_10_ ESC MIC to molecular markers in 124 *N. gonorrhoeae* isolates**

|  | **Ceftriaxone** | | |  | **Cefixime** | | |
| --- | --- | --- | --- | --- | --- | --- | --- |
| **Molecular marker** | **Coefficient** | **95% CI** | **p-value** |  | **Coefficient** | **95% CI** | **p-value** |
| Mosaic *penA* | 1.1369 | 0.69-1.58 | **<0.0001** |  | 2.247 | 1.73-2.77 | **<0.0001** |
| PBP2 A501T | 0.8172 | 0.34-1.29 | **<0.001** |  | 0.6689 | 0.11-1.22 | **0.019** |
| PBP2 A501V | 0.4962 | 0.14-0.85 | **0.007** |  | 0.4311 | 0.02-0.84 | **0.038** |
| PorB1B GA120KD | 0.2675 | -0.01-0.55 | 0.063 |  |  |  |  |
| PorB1B G213S/Y | 0.7056 | 0.28-1.13 | **0.001** |  | 0.6564 | 0.16-1.15 | **0.010** |

Multiple linear regression analysis was performed to determine the relationship of log_10_ (ceftriaxone/ cefixime MIC) as the dependent variable to the presence of gene mutations. The regression models were built from a preliminary analysis that included all related gene mutations, followed by backward, stepwise approach.

**Supplementary Table S5. Multiple regression analysis of the correlation of log_10_ azithromycin MIC to molecular markers in 124 *N. gonorrhoeae* isolates**

| **Molecular marker** | **Coefficient** | **95% CI** | **p-value** |
| --- | --- | --- | --- |
| 23s rRNA A2059G | 4.5238 | 3.97- 5.08 | **<0.0001** |
| MtrR A39T | -0.9516 | -1.49- -0.41 | **<0.001** |
| MtrR A40D | -1.2709 | -1.85- -0.69 | **<0.0001** |
| MtrR G45D | -0.9651 | -1.46- -0.47 | **<0.001** |
| MtrR H105Y | -0.7602 | -1.22- -0.3 | **0.001** |

Multiple linear regression analysis was performed to determine the relationship of log_10_ (azithromycin MIC) as the dependent variable to the presence of gene mutations. The regression models were built from a preliminary analysis that included all related gene mutations, followed by backward, stepwise approach.

**Supplementary Table S6. Linear regression analysis of ciprofloxacin resistance in 124 *N. gonorrhoeae* isolates**

| **Molecular marker** | **Coefficient** | **95% CI** | **p-value** |
| --- | --- | --- | --- |
| parC 85 mutations | 1.64 | 0.48-2.79 | **0.006** |
| parC 86 mutations | 1.59 | 0.55-2.63 | **0.003** |
| parC 87 mutations | 1.90 | 1.17-2.62 | **<0.0001** |
| parC 88 mutations | 1.05 | 0.42-1.68 | **0.001** |
| parC 89 mutations | 1.70 | 0.11-3.28 | **0.036** |
| parC 91 mutations | 1.35 | 0.28-2.41 | **0.014** |
| GyrA 91 mutations | 2.66 | 0.37-4.95 | **0.023** |
| gyrA 92 mutations | 1.33 | 0.56-2.10 | **0.001** |

Multiple linear regression analysis was performed to determine the relationship of CIP MIC intervals as the dependent variable to the presence of gene mutations. CIP MICs ranging from 0.004 to ≥ 32 mg/L corresponded to incremental MIC values of 1 to 8, respectively. The regression models were built from a preliminary analysis that included all related gene mutations, followed by backward, stepwise approach.
