# Supplementary figures and images for: Shanghai Neisseria gonorrhoeae Isolates Exhibit Resistance to Extended-Spectrum Cephalosporins and Clonal Distribution
Source: Front Microbiol. 2020 Oct 6;11:580399. doi: 10.3389/fmicb.2020.580399 (PMC7573285; doi:10.3389/fmicb.2020.580399)

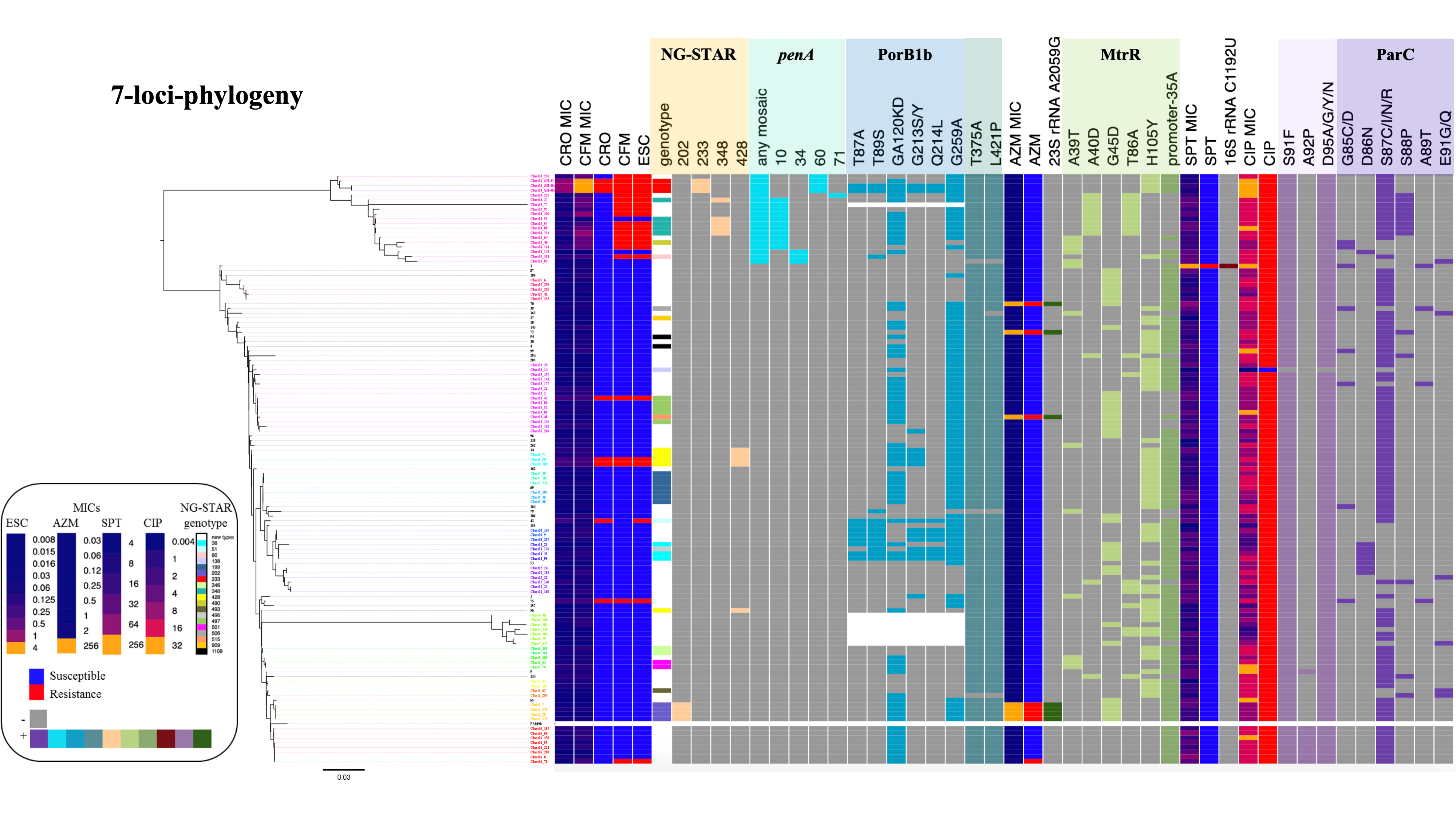

Supplement: Supplementary file 2 [file Image_1.JPEG]
